# Supplementary material for: The seroprevalence of SARS-CoV-2-specific antibodies in Australian children: A cross-sectional study
Source: PLoS One. 2024 Sep 18;19(9):e0300555. doi: 10.1371/journal.pone.0300555 (PMC11410239; doi:10.1371/journal.pone.0300555)
Supplement: S1 Table — (DOCX) [file pone.0300555.s001.docx]

**S1 Table:** Crude SARS-CoV-2 spike and nucleocapsid antibody seropositivity in the Australian children undergoing an anaesthetic procedure through PAEDS hospitals, with select underlying medical conditions in comparison to those without underlying medical conditions.

|  | | | S antibody n/N % | N-antibody n/N % |
| --- | --- | --- | --- | --- |
|  | **n** | **%**  **n/728** |  |  |
| **Participants without an underlying medical condition** | **1315/2043** |  | 1187/1315 (90.3) | 878/1315 (66.8) |
| **Total participants with 1 or more underlying medical condition^a^** | **728/2043** |  | 644/727 (88.6) | 730/728 (59.1) |
|  |  |  |  |  |
| Airway/chest disease | 213 | 29.2 | 184/213 (86.4) | 130/213 (61.0) |
| Cardiac/heart disease | 101 | 13.9 | 89/101 (88.1) | 60/101 (59.4) |
| Neurological/neuromuscular disease | 107 | 14.7 | 99/107 (92.5) | 66/107 (61.7) |
| Gastrointestinal disease | 97 | 13.3 | 84/97 (86.6) | 59/97 (60.8) |
| Genetic disorder | 71 | 9.8 | 63/71 (88.7) | 27/71 (38.0) |
| Kidney disease | 46 | 6.3 | 39/46 (84.8) | 28/46 (60.9) |
| Bone and joint disease | 43 | 5.9 | 38/43 (88.4) | 8/43 (65.1) |
| Developmental/behavioural disorder | 26 | 3.6 | 24/25 (96.0) | 17/26 (65.4) |
| Endocrine disease; non-diabetes | 15 | 2.1 | 12/15 (80.0) | 6/15 (40.0) |
| Eye disease/disorder | 11 | 1.5 | 9/11 (81.8) | 5/11 (45.5) |
| Blood disorders | 14 | 1.9 | 14/14 (100.0) | 9/14 (64.3) |
| Allergy/skin | 14 | 1.9 | 13/14 (92.9) | 10/14 (71.4) |
| Oncology history | 13 | 1.8 | 13/13 (100.0) | 9/13 (69.2) |
| Liver disease | 3 | 0. | 3/3 (100.0) | 2/3 (66.7) |
| Diabetes | 8 | 1.1 | 6/8 (75.0) | 4/8 (50.0) |
| Ear disease/disorder | 8 | 1.1 | 6/8 (75.0) | 5/8 (62.5) |
| Vascular, not intracranial | 5 | 0.6 | 5/5 (100.0) | 4/5 (80.0) |

^a^Total number of chronic medical conditions adds to more than 728 as some children had multiple conditions
